# Recurrent Turnover of Chromosome-Specific Satellites in *Drosophila*

**DOI:** 10.1093/gbe/evu104

**Published:** 2014-05-19

**Authors:** Miguel Gallach

**Affiliations:** Center for Integrative Bioinformatics Vienna (CIBIV), Max F. Perutz Laboratories, University of Vienna and Medical University of Vienna, Austria

**Keywords:** dosage compensation, speciation, X chromosome, dot chromosome

## Abstract

Repetitive DNA are DNA sequences that are repeated multiple times in the genome and normally considered nonfunctional. Several studies predict that the rapid evolution of chromosome-specific satellites led to hybrid incompatibilities and speciation. Interestingly, in *Drosophila*, the X and dot chromosomes share a unique and noteworthy property: They are identified by chromosome-specific binding proteins and they are particularly involved in genetic incompatibilities between closely related species. Here, I show that the X and dot chromosomes are overpopulated by certain repetitive elements that undergo recurrent turnover in *Drosophila* species. The portion of the X and dot chromosomes covered by such satellites is up to 52 times and 44 times higher than in other chromosomes, respectively. In addition, the newly evolved X chromosome in *D. pseudoobscura* (the chromosomal arm XR) has been invaded by the same satellite that colonized the ancestral X chromosome (chromosomal arm XL), whereas the autosomal homologs in other species remain mostly devoid of satellites. Contrarily, the Müller element F in *D. ananassae*, homolog to the dot chromosome in *D. melanogaster*, has no overrepresented DNA sequences compared with any other chromosome. The biology and evolutionary patterns of the characterized satellites suggest that they provide both chromosomes with some kind of structural identity and are exposed to natural selection. The rapid satellite turnover fits some speciation models and may explain why these two chromosomes are typically involved in hybrid incompatibilities.

## Introduction

*Drosophila*’s X and dot chromosomes (Müller elements A and F, respectively) share a unique and noteworthy property: They are identified by chromosome-specific binding proteins. Thus, the dosage compensation complex (DCC) uniquely binds the X chromosome in males (Straub and Becker 2007) whereas painting of fourth (POF) binds the polytenic (euchromatic) portion of the dot chromosome in both sexes (Larsson et al. 2001, 2004). How these proteins identify their target chromosome is poorly understood, although important progress has been made, in particular, regarding dosage compensation. According to a widely accepted model, the DCC is recruited in males to a limited number of high-affinity sites distributed across the X chromosome (also known as high-affinity chromatin entry sites; Alekseyenko et al. 2008; Straub et al. 2008), from where the DCC epigenetically spreads in *cis* to the rest of the chromosome. A GA-rich DNA sequence motif seems to be targeted in high affinity DCC binding sites (Alekseyenko et al. 2008) and, most notable, functionally conserved between distantly related *Drosophila* species (Alekseyenko et al. 2013).

An important caveat of this model is that the GA-rich DNA sequence motif mostly occurs outside the known DCC binding sites and its genome distribution pattern cannot predict X chromosome targeting (Conrad and Akhtar 2011). This strongly suggests that additional DNA sequence elements (Gallach et al. 2010) and/or long-range chromatin context (Conrad and Akhtar 2011) are important for DCC recruitment. On the other hand, several studies seem incompatible with the idea that a recognition element is conserved among *Drosophila* species. Hence, population genetic studies have showed that several components of the DCC, as well as several X chromosome entry sites, are most likely evolving under positive selection (Levine et al. 2007; Rodriguez et al. 2007; Bachtrog 2008). In addition, the functional conservation of this motif also seems incompatible with studies showing that the DCC fails to identify the X chromosome in male hybrids resulting from crosses between closely related species (Pal Bhadra et al. 2006; Chatterjee et al. 2007). These results support the hypothesis that failures in the dosage compensation system in hybrids may contribute to speciation (Orr 1989; Rodriguez et al. 2007). A recent study suggests that a disruption of the species-specific epistatic interactions between chromatin-remodeling factors and the X chromosome may cause a defect in the X-chromatin structure in the hybrid, one consequence of which is the mislocalization of the DCC (Barbash 2010). I think that this model may reconcile the conflicting observations: If a higher order architecture specific to the X chromosome is a prior determining factor on chromosome identification (Conrad and Akhtar 2011), functionally conserved DNA sequence motifs will be targeted by the DCC within species but not in the hybrids, where the chromatin structure would be distorted and unrecognizable (Barbash 2010). Unfortunately, it is not known whether POF fails to localize the dot chromosome in *Drosophila* hybrids, as described for the DCC and the X chromosome. This experiment remains to be done and will certainly shed light on the roles of POF in the speciation process.

Noncoding repetitive DNA has the ability to adopt specific folding structures capable of attracting chromatin remodeling proteins (Podgornaya et al. 2013). This property makes repetitive DNA a potential carrier of a “chromatin folding code” (Vogt 1990; Podgornaya et al. 2013), which may help cells identify chromosomes or specify chromosome territory rearrangements (Podgornaya et al. 2013). Currently, the role of repetitive DNA elements has become a major interest among evolutionary biologists as recent studies have shown that species-specific interactions between chromatin remodeling proteins and repetitive DNA elements are disrupted in hybrids (Brideau et al. 2006; Bayes and Malik 2009; Ferree and Barbash 2009). According to a general model, sets of satellites and their corresponding binding proteins will evolve independently from those of different species (Maheshwari and Barbash 2011; Ferree and Prasad 2012). Thus, lineage-specific changes in the structure, sequence, or localization of certain repetitive DNA elements may originate genetic conflicts between closely related species or populations, eventually resulting in hybrid incompatibilities (Henikoff et al. 2001; Brideau et al. 2006; Bayes and Malik 2009; Ferree and Barbash 2009; Barbash 2010; Maheshwari and Barbash 2011; Ferree and Prasad 2012). Interestingly, satellites in the X-heterochromatin and dot chromosomes are also involved in such processes in *Drosophila* (Braverman et al. 1992; Brideau et al. 2006; Bayes and Malik 2009; Ferree and Barbash 2009).

Despite the aforementioned evidence, the potential of repetitive DNA elements to explain both chromosome-specific targeting and hybrid incompatibility remains unexplored (Maheshwari and Barbash 2011). In an attempt to do so, I have applied a DNA sequence analysis called oligonucleotide profiling (Arnau et al. 2008) to several *Drosophila* species, covering the genus. I describe the existence of different repetitive DNA sequences that overpopulate the euchromatin of the X and dot chromosomes. The genome distribution of these sequences and their evolutionary patterns agrees with speciation models and suggests that they may provide these two chromosomes with a structural identity.

## Results and Discussion

**Fig. 1.—:**
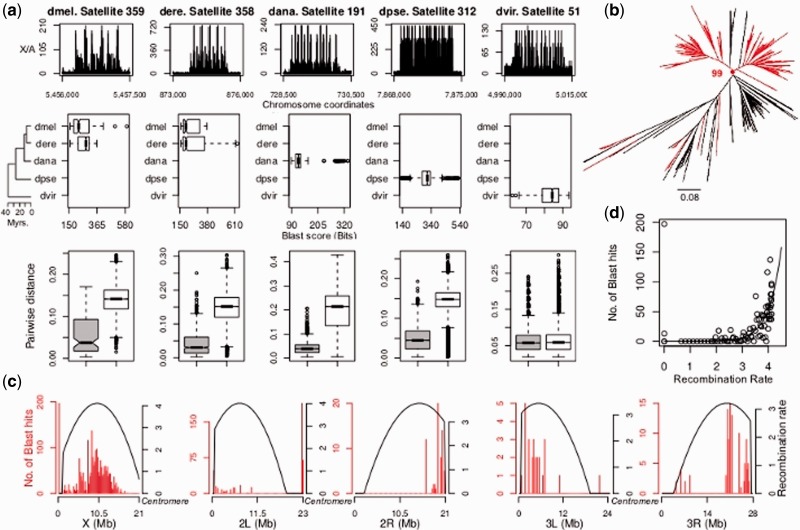
Properties of the satellites overpopulating the X chromosomes. (*a*) Each satellite species shows a characteristic X/A profile (first row), restricted species distribution (second row), and undergoes concerted evolution (third row). Third row: The distance between copies of the same locus (gray) is lower than that of different loci (white). *P* < 2.2 × 10^−16^ for each pair comparison, using Wilcoxon rank-sum test. (*b*) Reconstructed ML tree for the dere. Satellite 358 copies found in *Drosophila erecta* (red) and *D. melanogaster* genomes (black). (*c*) BLAST hits found for dmel. Satellite 359 and recombination rates in *D. melanogaster*, computed for nonoverlapping windows of 250 kb. (*d*) Correlation between number of BLAST hits and recombination rate. The black line corresponds to the fitted exponential function: Number of hits = e^(−4.56 + 2.14*recombination rate)^.

I performed oligonucleotide profiling (Arnau et al. 2008) to compute relative 13-mer frequencies between pairs of chromosomes in *D. melanogaster*, *D. erecta*, *D. ananassae*, *D. pseudoobscura,* and *D. virilis* species. The relative frequency is a normalized quotient that indicates how often a k-mer occurs in one chromosome compared with another (see Materials and Methods). When performed for each consecutive k-mer occurring in a chromosome, a chromosome-wide k-mer (oligonucleotide) profile is generated. The intraspecific comparison between the X chromosome and the autosomes generates a steep profile along the X chromosome (i.e., X/A profile), with a plethora of X/A values higher than 1, where X/A = *i* means that the 13-mer is *i*-times more frequent in the X chromosome than in the autosomes (fig. 1*a*). As expected (Gallach et al. 2007), the comparison between autosomes (A/A profiles) generated a flat profile around A/A = 1, indicating similar 13-mer frequencies among them (not shown). I manually scanned the X/A profiles to detect clusters of overrepresented 13-mers along the X chromosome and found typical clusters spanning from approximately 1 to approximately 20 kb and reaching X/A values between 130 and 720, depending on the species (fig. 1*a*). Interestingly, the structure of each cluster revealed an internal repetitive pattern generated by repeats arranged in tandem, which I further characterized (Materials and Methods).

I characterized three repetitive units, or monomers, in *D. melanogaster*, the most frequent one defined as a 359-bp DNA sequence (dmel. Satellite 359), which, according to RepBase and Censor (Kohany et al. 2006), are related to the 1.688 satellite related repeat (DiBartolomeis et al. 1992; supplementary fig. S1, Supplementary Material online). A few euchromatic loci containing several copies of the satellite were already described in the literature (Waring and Pollack 1987; DiBartolomeis et al. 1992). However, I found 2,655 related sequences (BLAST hit *E* < 10^−4^) dispersed in the X chromosome and, remarkably, the percentage of this chromosome covered by the satellite is 45 times higher than the autosomes (table 1). Interestingly, it has been shown that this satellite influences the chromatin structure of the chromosomal domain where it is located (Benos et al. 2000). Therefore, this satellite not only provides the X chromosome with a chromosome-wide DNA sequence identity, but, in addition, the X chromosome may exhibit a differentiated long-range chromatin structure compared with other chromosomes in which this satellite is scarce.

**Table 1:** Satellite Presence^a^ in the Species Where They Have Been Described

	Müller Element (corresponding name in *Drosophila melanogaster*)					
	A (X)	B (2L)	C (2R)	D (3L)	E (3R)	X/A^b^
*D. melanogaster*	2,655 (2.91)	577 (0.41)	82 (0.06)	44 (0.03)	158 (0.11)	45
*D. erecta*	2,087 (2.69)	186 (0.16)	74 (0.072)	157 (0.17)	183 (0.24)	20
*D. ananassae*	468 (0.28)	8 (0.003)	30 (0.015)	99 (0.071)	9 (0.003)	52
*D. pseudoobscura*	333 (0.26)	447 (0.43)^c^	10 (0.015)	1,105 (1.15)	98 (0.067)	7; 32
*D. virilis*	1,525 (0.24)	96 (0.014)	54 (0.007)	20 (0.003)	198 (0.023)	35

^a^Number of BLAST hits and the percentage of the chromosome they cover (in brackets) are given. All the characterized families were used as query.

^b^Percentage of the X chromosome divided the percentage of the autosomes covered by the satellites, averaged for all comparisons. The first X/A value provided for *D. pseudoobscura* corresponds to XL/A and the second to XR/A.

^c^The scaffold Ch4_group3 contains 84% of all the hits in this chromosome whereas, according to its length, it is expected to contain 43% of them. The percentage of the Müller element B covered by the satellites is actually 0.001% if we exclude this scaffold.

I further characterized the DNA sequences generating typical cluster profiles in the other species. These sequences also consist of dispersed copies of tandem repeats, most of which have never been described before (supplementary fig. S1, Supplementary Material online). These satellites differ in sequence, length and copy number among species, therefore revealing a recurrent turnover during the evolution of *Drosophila* species (fig. 1*a*). In addition, the portion of the X chromosome covered by these satellites is also remarkably higher than in the autosomes (table 1). Because dere. Satellite 358 shows a similarity of 79% to the 1.688 satellite related repeat, BLAST searches of this element found significant hits in *D. melanogaster’*s genome (fig. 1*a*). To determine whether the dere. Satellite 358 and dmel. Satellite 359 copies are orthologous, I compiled full-length copies of dere. Satellite 358 detectable in *D. melanogaster* and *D. erecta* genomes (146 and 400 copies, respectively) and reconstructed a maximum likelihood (ML) tree from the multiple alignment. The ML tree clusters copies from the same species together (bootstrap value: 99; fig. 1*b*), indicating that the satellites found in each species represent, most likely, different colonizing episodes from different founder elements, consistent with the turnover observed in the other species. The recurrent change in sequence, location, and copy number of this type of satellite is in agreement with speciation studies in *Drosophila* (Brideau et al. 2006; Bayes and Malik 2009; Ferree and Barbash 2009), and may also contribute to the fast expression divergence of the X-linked genes (Kayserili et al. 2012; Meisel et al. 2012).

Comparative genomics analyses revealed important aspects of the biology and evolutionary patterns of the satellites. As previously described for different heterochromatic satellite families in *Drosophila* (see Li 1997, and references therein), a recent study showed that several copies of the 1.688 satellite related repeat also undergo concerted evolution (Kuhn et al. 2012). Consistent with these observations, I found that satellite copies of the same locus share the same substitutions (supplementary fig. S2, Supplementary Material online), and the genetic distance between copies from the same locus is lower than the distance between copies from different loci (fig. 1*a*). Gene conversion and unequal crossing-over are probably the two most important mechanisms for the occurrence of concerted evolution (Li 1997). Unequal crossing-over is assumed to be the dominant mechanism driving concerted evolution of the heterochromatic satellites (Strachan et al. 1985; Li 1997), but it can cause deletions and duplications of the genes located between the repeats. Therefore, nonallelic gene conversion may be a better mechanism to explain the concerted evolution of the euchromatic satellites characterized in this study (Li 1997). Contrary to theoretical predictions (Charlesworth et al. 1986; Stephan 1986, 1989; Charlesworth et al. 1994; but see Smith 1976), I found a significant correlation between satellite abundance and recombination rate in *D. melanogaster* (fig. 1*c*). Such a correlation is exponential (fig. 1*d*), indicating that the satellites depend on the recombination rate to expand and remain in the chromosome, but above a certain threshold this dependence is weak. This result indicates that the molecular mechanisms driving the evolution of the euchromatic and heterochromatic satellites are most likely different.

Because autosomes experience lower recombination rates than those of X chromosomes in *Drosophila* (median: 2.78 and 3.32 cM/Mb, respectively; supplementary fig. S3, Supplementary Material online), recombination may explain the differences in satellite abundance between the X chromosome and the autosomes. To test this hypothesis, I plotted the satellite coverage as a function of the recombination rate (as in fig. 1*d*; not shown) and fitted the data to the exponential function: Coverage = e^(^^−11.82 + 1.98^*^recombination rate)^. After multiplying the recombination rate of the X chromosome by 4/3 to correct for differences in the effective population sizes between the X chromosome and the autosomes (Singh et al. 2005), I computed the ratio [coverage_X_/coverage_A_] = 25. In other words, the percentage of the X chromosome covered by these satellites is expected to be 25 times higher than the autosomes, and therefore, the differences in recombination rates between the X chromosomes and the autosomes may contribute to, but cannot satisfactorily explain, the overwhelming difference between the X chromosomes and the autosomes (45-fold; table 1).

Next, I took advantage of the chromosomal arrangement between the Müller elements A and D in *D. pseudoobscura* (chromosomal arms XL and XR, respectively) to test whether the satellite overabundance is just an intrinsic (historical) feature of the Müller element A or a convergent property of the X chromosomes. The ancestral autosome, Müller element D, fused to the X chromosome about 10 Ma (Richards et al. 2005), and this new X chromosome arm also recruits the DCC in this species (Marín et al. 1996). Remarkably, BLAST analysis shows that the chromosomal arm XR is overpopulated with the same DNA satellite as the chromosomal arm XL, whereas the autosomal homologs in the other species remain scarce of satellites (table 1).

**Fig. 2.—:**
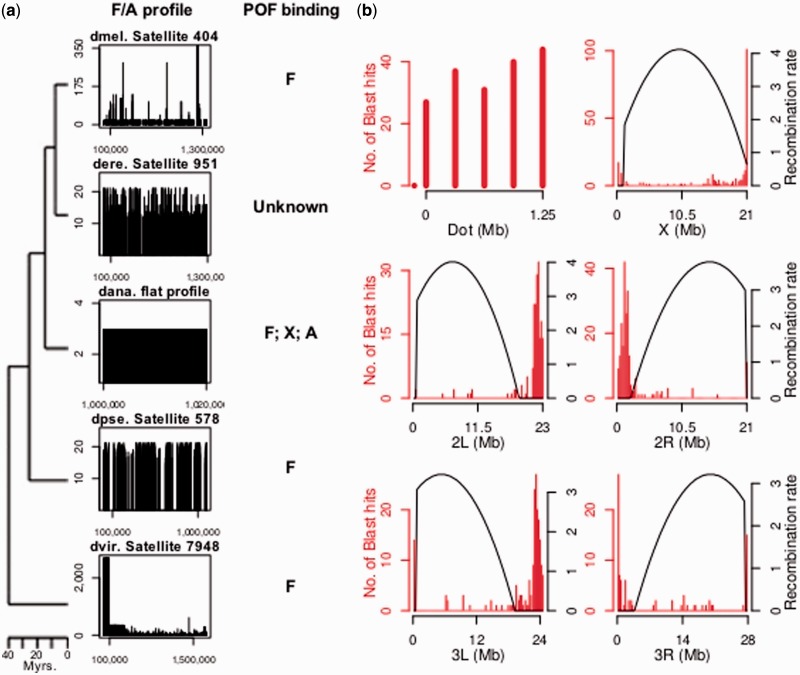
Properties of the satellites overpopulating the dot chromosomes. (*a*) Typical F/A profiles of the characterized satellites. POF binding pattern is given according to Larsson et al. (2004). POF binding is not specific to Müller element F in *Drosophila ananassae*, in which species no overrepresented 13-mers are found either. As *L*_F_ = 0.34 × *L*_A_ in *D. ananassae*, F/A = 2.94 when *k*_F_ = *k*_A_ (see Materials and Methods for details). (*b*) BLAST hits found for dmel. Satellite 404 and recombination rates in *D. melanogaster*, computed for nonoverlapping windows of 250 kb.

To test whether the correlation between chromosomal identity and satellite overpopulation is unique to the X chromosome, I further studied the dot chromosome. Oligonucleotide profiling of the Müller element F in *Drosophila* species reveals that these chromosomes also have higher relative amounts of repetitive elements (fig. 2*a* and table 2). Three of the characterized elements (dmel. Satellite 404, dere. Satellite 951, and dpse. Satellite 578) were identified as helitron-like sequences by RepBase. None of them corresponds to Dr.D or DINE-1, two previously described transposable elements (TEs) found at high frequency in the dot chromosome of *D. melanogaster* and *D. virilis* (Miklos et al. 1988; Locke et al. 1999; Slawson et al. 2006). BLAST searches did not detect the characterized elements outside the species in which they were described, indicating a recurrent turn-over (supplementary fig. S4, Supplementary Material online), as previously described for the X-specific satellites.

**Table 2:** Satellite Presence^a^ in the Species Where They Have Been Described

	Müller Element (corresponding name in *Drosophila melanogaster*)						
	F (4)	A (X)	B (2L)	C (2R)	D (3L)	E (3R)	F/(X + A)^b^
*D. melanogaster*	178 (1.31)	221 (0.096)	206 (0.085)	219 (0.093)	191 (0.072)	86 (0.028)	22
*D. erecta*	287 (3.23)	185 (0.09)	1,097 (0.55)	731 (0.39)	733 (0.38)	553 (0.26)	14
*D. pseudoobscura*	331 (2.78)	654 (0.29)	543 (0.19)	486 (0.24)	446 (0.24)	496 (0.18)	12
*D. virilis*	2,668 (3.11)	1,194 (0.03)	1,011 (0.02)	758 (0.017)	590 (0.013)	2,138 (0.13)	44

^a^Number of BLAST hits and the percentage of the chromosome they cover (in brackets) are given.

^b^Percentage of the dot chromosome divided the percentage of the other chromosomes covered by the satellites, averaged for all comparisons.

The correlation between TE overabundance and chromosomal identity of the dot chromosome could, however, have a simple explanation. Hence, in agreement with theory and data (Charlesworth et al. 1992, 1994; Bartolomé et al. 2002), nonrecombining regions in *D. melanogaster* accumulate most of the significant BLAST hits (fig. 2*b*), suggesting that the overabundance of dmel. Satellite 404 in the Müller element F may be due to the lack of recombination in this chromosome. However, recombination does not explain the pattern observed in other species. For instance, the polytenic dot chromosome in *D. virilis* is fully euchromatic and does recombine (Riddle and Elgin 2006), but contrary to theory, there is an exceptionally high overabundance of dvir. Satellite 7948 in this chromosome (table 2). On the other hand, the Müller element F in *D. ananassae* is fully heterochromatic and does not recombine (Schaeffer et al. 2008), and therefore, the overabundance of this kind of elements is expected. Contrary to expectation, there is no significant overrepresentation of 13-mers in this chromosome as compared with other chromosomes (fig. 2*a*). Notably though, the binding pattern of POF in *D. ananassae* is not specific to the Müller element F either, as it also binds the X chromosome in males and the autosomes under some conditions (Larsson et al. 2004). Altogether, the data show that there is also a correlation between repetitive elements overpopulation and chromosomal identity associated with the dot chromosome, and support the hypothesis that the overwhelming density of repetitive elements in this chromosome is selective advantageous (Slawson et al. 2006). Interestingly, TEs may harbor regulatory motifs which may be recruited in new chromosomal locations after their expansion throughout the genome, and this way, integrating genes into the same regulatory network (Feschotte 2008).

In summary, this study shows that the X and dot chromosomes are overpopulated with different types of satellites, which provide them with a specific DNA sequence composition and, probably, a unique, long-range, chromatin structure. The conclusion of this overabundance relies on the quality of the current genome assemblies. Therefore, some experimental validation (e.g., fluorescence in situ hybridization on polytenic chromosomes) would eventually be needed to confirm that the massive fold-enrichment in these two chromosomes is not due to a biased sampling of the assembled repeats. However, this potential caveat is very unlikely as one would expect an equal sampling bias across all chromosomes in each species, which is certainly not the case. The turnover of heterochromatic satellite families had been described a long time ago among *Drosophila* species, primates and rodents, but their function and implication in the speciation process have remained largely speculative (reviewed in Brutlag 1980). Currently, many studies show that highly repetitive DNA may carry out specific cellular functions (Podgornaya et al. 2013) and their rapid evolution may be involved in the speciation process. The recurrent turnover of the characterized satellites fits some speciation models, according to which, satellite divergence can serve as reproductive barriers between sibling species (summarized in Ferree and Prasad 2012). The discovery of these satellite species anticipates further functional and comparative studies that will help to understand the special biology and evolution of the X and dot chromosomes.

## Materials and Methods

### *Drosophila* Species and Chromosome Assemblies

Given the extent of the analysis, I choose five *Drosophila* species for this study. The species were chosen according to three criteria: They had to cover the whole genus, contain different karyotype configurations, and show newly evolved DCC and POF binding patterns. Release dmel_r5.26, dere_r1.3, dana_r1.3, dpse_r1.3, and dvir_r1.2 were downloaded from FlyBase (http://flybase.org/, last accessed May 26, 2014) and used as *D. melanogaster*’s, *D. erecta*’s, *D. ananassae*’s, *D. pseudoopscura*’s, and *D. virilis*’ genome sequence. Chromosomes were assembled according to Schaeffer et al. (2008).

### Characterization of the Repetitive Elements

Oligonucleotide profiling was applied as in Gallach et al. (2007). Briefly, the frequency of the consecutive 13-mers contained in the X chromosome was computed with UVWORD (Gallach et al. 2007; Arnau et al. 2008), and divided by their frequency in the autosomes. After normalizing for the chromosomal lengths, an X/A value was obtained for each 13-mer along the X chromosome. The relative frequency was computed as follows: For a 13-mer in the X chromosome, an X/A value was calculated as [*k*_X_ × *L*_A_]/[*k*_A_ × *L*_X_], where *k*_X_ and *k*_A_ are the number of occurrences of the 13-mer in the X chromosome and in the autosomes, and *L*_A_ and *L*_X_ are the lengths of the autosomes and the X chromosome, respectively. The same procedure was followed to obtain the F/A and A/A oligonucleotide profiles. Finally, I preferred the use of 13-mers because this length allows the detection of chromosome-specific sequences in *Drosophila* (Gallach et al. 2007). In addition, 13 is a prime number, and therefore, the search is less affected by the presence of simple repeats based on dinucleotides, trinucleotides, etc. (Gallach et al. 2007).

To characterize the repetitive unit, or monomer, I compiled the DNA sequences generating clusters of overrepresented 13-mers (i.e., X/A > 20). Therefore, repeats showing lower X/A values may still be undetected. Next, the sequences were given to Tandem Repeat Finder (Benson 1999) to identify the DNA sequence that maximized the alignment scores between the different monomers that could be defined in tandem. As the satellites found in each species are related to each other (e.g., dmel. Satellite 360 contains a partial inverted sequence of the other two satellites), I further used MEME to identify monomers of the same family. The monomer with maximum length was used as the representative copy for the satellite family and as the query sequence in further BLAST searches.

### Molecular Evolution Analysis

Multiple alignment of satellite copies was performed with MAFFT (Katoh and Standley 2013) and corrected by hand with Jalview (Waterhouse et al. 2009). The hamming distance between aligned copies was calculated with the program distmat, included in the JEMBOSS software suite (Carver and Bleasby 2003). Copies located within 1 kb of each other were considered to belong to the same locus. The ML tree was computed with IQ-TREE (Minh et al. 2013), which automatically selects the best-fit model according to the Bayesian information criterion.

### Satellite Density and Recombination Rate

*Drosophila melanogaster* chromosome sequences were split into nonoverlapping windows of 250 kb, and the number of BLAST hits and the averaged recombination rate were computed for each of them. Recombination rates were calculated for each window with the Recombination Rate Calculator (http://petrov.stanford.edu/RRC_scripts/RRC-open-v2.2.1.pl, last accessed May 26, 2014) and the median recombination rates for the X chromosome and the autosomes were computed from them.

All the analyses were carried out with the R statistical computing software (http://www.r-project.org/, last accessed May 26, 2014). Satellite alignments are available upon request to the author

## Supplementary Material

Supplementary figures S1–S4 are available at *Genome Biology and Evolution* online (http://www.gbe.oxfordjournals.org/).

Supplementary Data
