# Supplementary material for: Recurrent Turnover of Chromosome-Specific Satellites in Drosophila
Source: Genome Biol Evol. 2014 May 19;6(6):1279–86. doi: 10.1093/gbe/evu104 (PMC4079201; doi:10.1093/gbe/evu104)
Supplement: Supplementary Data [file supp_6_6_1279__index.html]

Recurrent turnover of chromosome-specific satellites in Drosophila. — Recurrent Turnover of Chromosome-Specific Satellites in Drosophila — Supplementary Data 

# Recurrent Turnover of Chromosome-Specific Satellites in *Drosophila*

## Supplementary Data

files

**Files in this Data Supplement:**

- Supplementary Data - zip file
