# Supplementary material for: Recurrent Turnover of Chromosome-Specific Satellites in Drosophila
Source: Genome Biol Evol. 2014 May 19;6(6):1279–86. doi: 10.1093/gbe/evu104 (PMC4079201; doi:10.1093/gbe/evu104)

**Supplementary Fig. 1.** Description of the Repeat Class elements (Repbase name in brackets) showing similarity with the characterized satellites. Red blocks represent the portion of the characterized satellite that can be aligned with the corresponding Repeat Class element.

| Satellite | Repeat Class | SGV Plot |
|---|---|---|
| dmel. Satellite 358 | 1.688 satellite related (SAR2_DM) | |
| dmel. Satellite 359 | 1.688 satellite related (SAR_DM) | |
| dmel. Satellite 360 | 1.688 satellite related (XD_MR) | |
| dere. Satellite 358 | 1.688 satellite related (SAR_DM) | |
| dere. Satellite 360 | 1.688 satellite related (SAR2_DM) | |
| dana. Satellite 191 | No hit | No hit |
| dpse. Satellite 312 | No hit | No hit |
| dvir. Satellite 51 | No hit | No hit |
| dmel. Satellite 404 | Helitron | |
| dere. Satellite 951 | Helitron | |
| dpse. Satellite 578 | Helitron | |
| dvir. Satellite 7948 | Politon, Jokey, Copia, L1, Gypsy | |

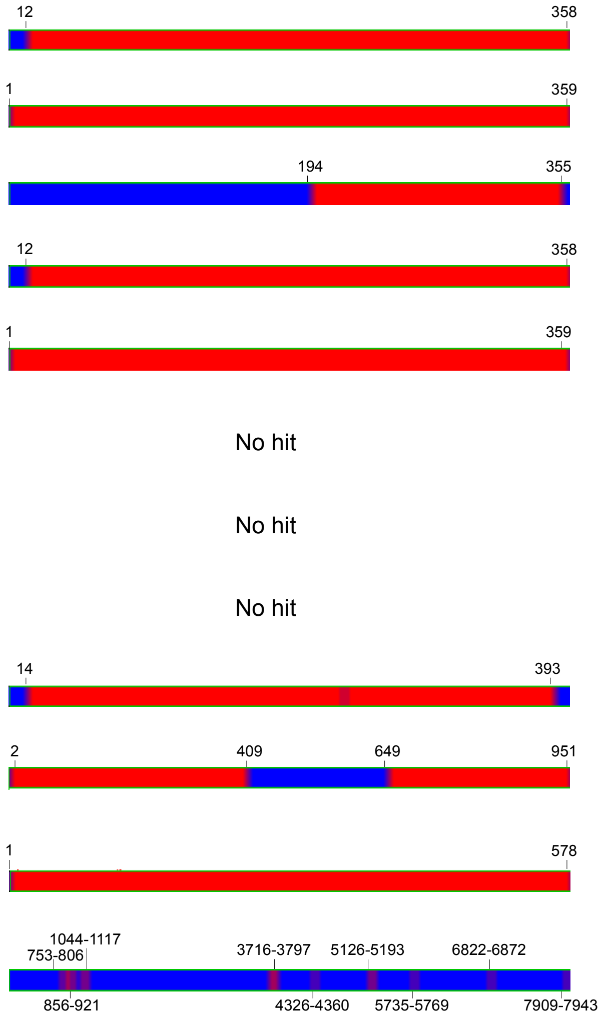

**Supplementary Fig. 2.** Copies of the same locus share the same nucleotide substitutions. Loci L3, L4 and L5 characterized in *D. erecta* are shown in this example. These loci contain four, five, and four copies, respectively, of the dere. Satellite 358 in the minus strand of the X chromosome. The same colors used in the schematic to denote the loci (left side of the figure) are used to frame the corresponding satellite copies in the alignment. Number 47 makes reference to the position in the original alignment. Black bars indicate alignment sites showing shared substitution among copies of the same locus.

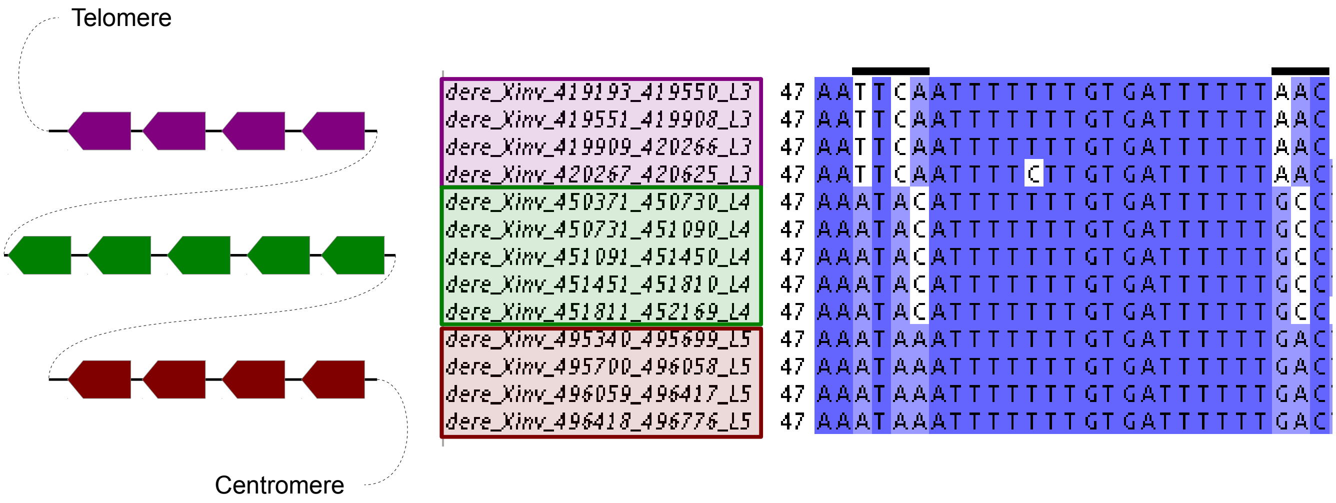

**Supplementary Fig. 3.** Comparison between the X chromosome's and autosomes' recombination rates.

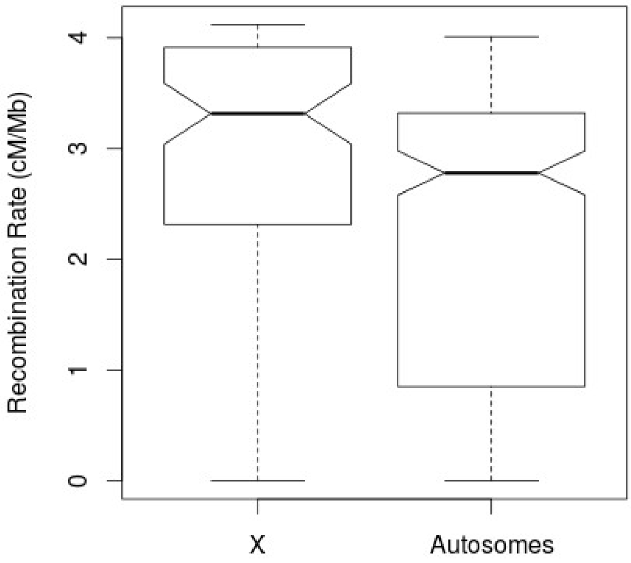

**Supplementary Fig. 4.** BLAST score distribution of the significant hits ($E < 10^{-4}$) found in each *Drosophila* species.

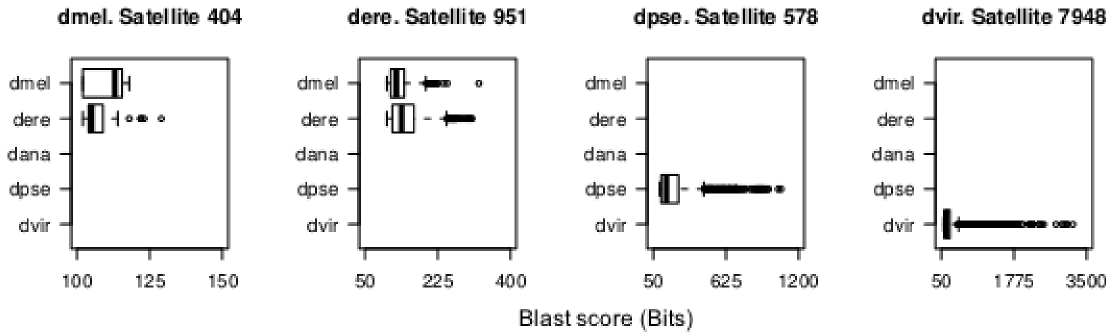

Supplement: Supplementary Data [file supp_evu104_suppl_data.zip › Gallach_Supplementary_information.pdf]
